# Molecular cloning and *in-silico* characterization of high temperature stress responsive *pAPX* gene isolated from heat tolerant Indian wheat cv. Raj 3765

**DOI:** 10.1186/1756-0500-7-713

**Published:** 2014-10-10

**Authors:** Jasdeep Chatrath Padaria, Harinder Vishwakarma, Koushik Biswas, Rahul Singh Jasrotia, Gyanendra Pratap Singh

**Affiliations:** Biotechnology and Climate Change Laboratory, National Research Centre on Plant Biotechnology, Pusa Campu, New Delhi, 110012 India; Division of Genetics, IARI, Pusa Campu, New Delhi, 110012 India

**Keywords:** Cloning, *In-silico*, Peroxisomal ascorbate peroxidase, Homology modeling, Expression, cv. Raj3765

## Abstract

**Background:**

Heat stress leads to accelerated production of reactive oxygen species (ROS) which causes a huge amount of oxidative damage to the cellular components of plants. A large number of heat stress related genes as HSPs, catalases, peroxidases are overexpressed at the time of stress. A potent stress responsive gene peroxisomal ascorbate peroxidase (*TapAPX*) obtained from heat stress (42°C) responsive subtractive cDNA library from a thermo tolerant wheat cv. Raj3765 at anthesis stage was cloned, characterized and its role was validated under heat stress by proteomics and *in-silico* studies. In the present study we report the characterization at molecular and *in-silico* level of peroxisomal *TapAPX* gene isolated from heat tolerant wheat cultivar of India.

**Results:**

qPCR studies of *TapAPX* gene displayed up to 203 fold level of expression at 42°C heat stress exposure. A full length cDNA of 876 bp obtained by RACE deduced a protein of 292 amino acid residues which gives a complete 3D structure of *pAPX* by homology modeling. *TapAPX* cDNA was cloned in expression vector pET28 (a+) and the recombinant protein over-expressed in *E. coli* BL21 showed highest homology with APX protein as deduced by peptide mass fingerprinting.

**Conclusions:**

*TapAPX* gene from wheat cv Raj3765 has a distinct role in conferring thermo tolerance to the plants and thus can be used in crop improvement programmes for development of crops tolerant to high temperature.

**Electronic supplementary material:**

The online version of this article (doi:10.1186/1756-0500-7-713) contains supplementary material, which is available to authorized users.

## Background

Heat stress in plants produces large number of Reactive Oxygen Intermediates (ROIs) like superoxide ion (O^2-^), hydroxide ion (OH^-^), singlet oxygen (O_2_^*^), H_2_O_2_ etc. excess of which can lead to damage of plant cells. Among these ROS (Reactive Oxygen Species), H_2_O_2_ can accumulate in cells to toxicity levels because of its high stability. A number of cellular enzymes as superoxide dismutase, monodehydroascorbate reductase, glutathione reductase and ascorbate peroxidase are produced by the cell to get rid of high level of H_2_O_2_. Ascorbate peroxidase plays a leading role in removing ROIs in ascorbate-glutathione cycle [1]. Four types of *APX* isoforms have been identified based on the phylogenetic analysis: cytoplasmic *APX*1 and *APX2*, chloroplastic *APX* and membrane bound *APX* [2]. Upregulation of *APX* genes was observed under abiotic stress conditions in rice, white birch and *Suaeda salsa* [3–5] and *APX* has also been reported in different food crops like pea, cayenne pepper, grape [6–8]. *APX* thus has a distinct role in conferring tolerance to plants against abiotic stress.

In the present study, the coding sequence of peroxisomal or glyoxisomal Ascorbate peroxidase (*TapAPX*) gene [Genbank:JX126968] (http://www.ncbi.nlm.nih.gov/) from a heat tolerant cultivar Raj3765 [9] of Indian bread wheat (*Triticum aestivum* L.) designated as *TapAPX* was cloned and characterized. The *TapAPX* gene was subcloned in pET-28a and transformed in *E. coli* for heterologous protein expression studies. The expressed protein *Ta*pAPX was confirmed by SDS-PAGE analysis, western blotting and peptide mass fingerprinting. The over expression of *Ta*pAPX protein in bacterial system under heat stress was validated and the over-expressed protein was purified using Ni-NTA His-tag purification column for further proteomics studies. Homology search based modeling was performed to deduce a three dimensional (3-D) structure of the protein. The refined structure of generated *Ta*pAPX was confirmed with its template structure followed by identification of its active site residues. The functional correlation and interaction between the *Ta*pAPX and its substrate H_2_O_2_ was validated by docking analysis.

## Results

### Lipid peroxidation assay, subtracted cDNA library preparation and functional annotation

Estimation of lipid peroxidation was done for the leaf samples collected from plants subjected to heat stress for different time intervals. Non-specific absorbance of the extract at 600 nm was subtracted from the 532 nm readings. The MDA (malondialdehyde) concentration in nmol/g dry weight (nmol/gDW) was calculated. Samples of heat susceptible cv. HD 2967 subjected to heat stress of 37°C and 42°C for 30 min to 6 h, showed statistical significant changes as compared to control (Table 1), an increase in MDA concentration in the range of 40.56 nmol/gDW to 90.95 nmol/gDW and 41.99 nmol/gDW to 108.56 nmol/gDW respectively was observed. Whereas, samples of heat tolerant cv Raj 3765 subjected 37°C and 42°C heat stress for 30 min to 6 h showed increase as well as decrease in MDA concentration in comparison to control. MDA concentration in heat stressed samples of cv Raj 3765 varied from 64.54 nmol/gDW to 106.67 nmol/gDW (37°C) and 44.90 nmol/gDW to 112.28 nmol/gDW (42°C). To identify differentially expressed heat stress responsive genes in wheat cv.Raj 3765 plants at anthesis stage, 42°C. Heat stress responsive subtractive cDNA libraries were constructed in pGEM-T easy vector. A total of 545 clones were obtained from forward EST (Expressed Sequence Tags) library and colony PCR using T7/SP6 primers confirmed 253 clones to have insert size ranging from 250 to 1500 bp. Sequencing of randomly selected 250 clones confirmed a total number of 204 high quality ESTs (http://www.ebi.ac.uk/ena/) after removal of vector (NCBI/vecscreen) and adaptor sequences. After assembly of 204 ESTs [ENA:HG314154-HG314357], 149 unigenes containing 45 contigs and 104 singletons were obtained. Similarity analysis of 149 unigene sets by BLASTX search confirmed annotation of 101 unigenes where 48 EST sequences showing no hit (Additional file 1: Table S1).

**Table 1:** **Absolute content of MDA (Malondialdehyde) in nmol/g dry weight showing significant changes**

Time given for heat stress (in h)	HD 2967, 37°C A _532_-A _600_	Raj 3765, 37°C time A _532_-A _600_	HD 2967, 42°C time A _532_-A _600_	Raj 3765, 42°C time A _532_-A _600_
Control	38.68 ± 0.13a	95.56 ± 0.68ab	38.68 ± 0.13a	95.56 ± 0.68ab
½	90.95 ± 1.90b	106.15 ± 1.11a	99.86 ± 2.65b	44.90 ± 1.43d
1	87.06 ± 0.47bc	85.84 ± 6.17b	103.91 ± 3.29b	112.28 ± 0.26a
2	80.34 ± 0.42c	64.54 ± 0.50c	108.56 ± 7.14b	82.33 ± 1.64b
4	40.56 ± 2.88a	106.67 ± 1.14a	69.99 ± 1.09c	69.59 ± 1.15c
6	52.82 ± 0.30d	64.58 ± 0.82c	41.99 ± 5.51a	108.17 ± 7.39a

Mean values having the same letter in each column are not significantly different at P = 0.05 (Tukey test) (n = 3).

### Real time quantification for *TapAPX*gene

**Figure 1:**
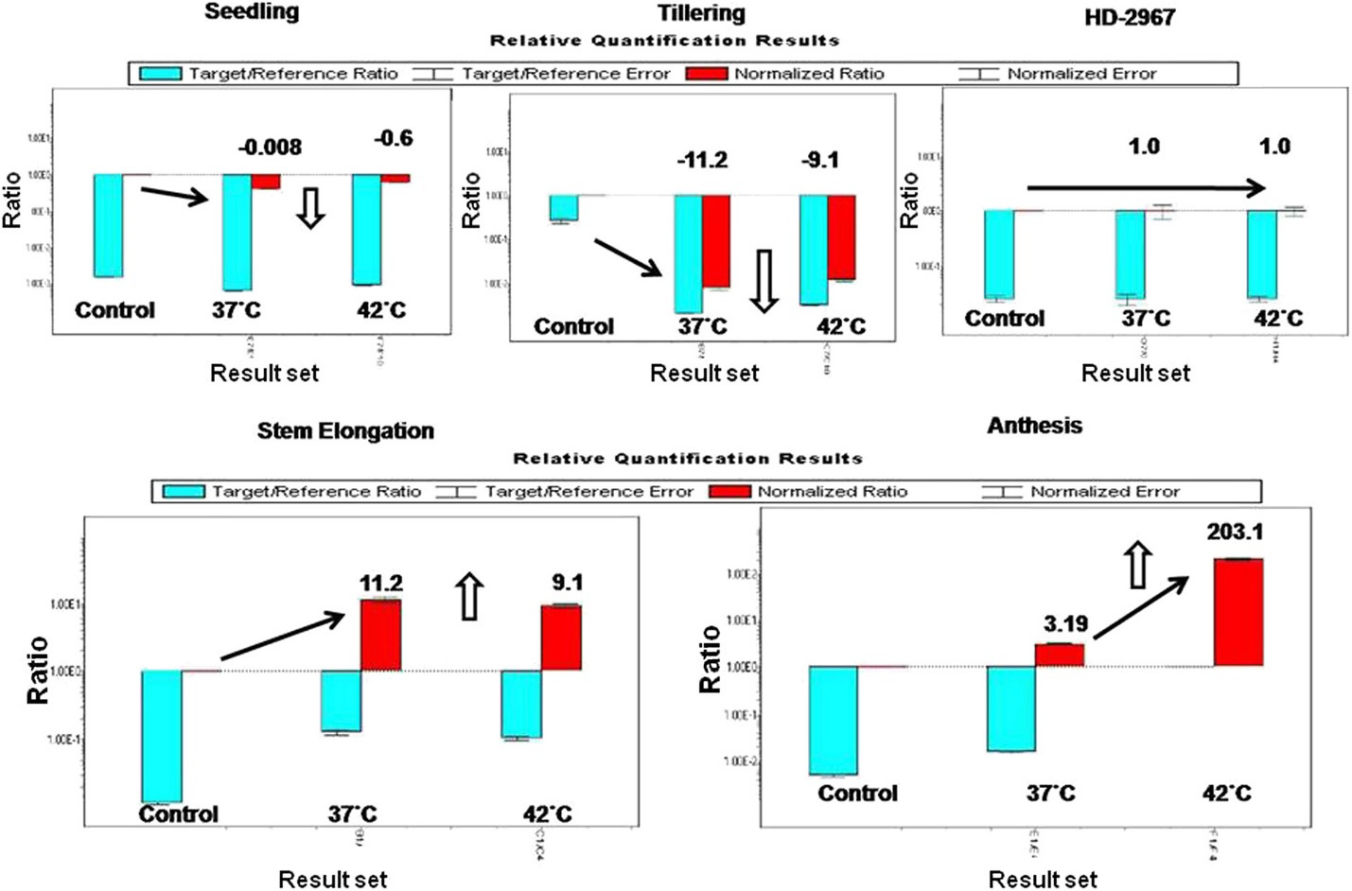
**qPCR profiling of** ***TapAPX*** **(peroxisomal ascorbate peroxidase) gene at different developmental stages in thermo-tolerant wheat cv. Raj 3765 and at anthesis stage in susceptible cultivar of wheat HD 2967.**

Functional annotation of obtained EST sequences identified a number of genes (5.38%) expressed in response to abiotic and biotic stress in wheat cv. Raj 3765. A transcript with 720 bp showed highest similarity (97%) with *APX* gene in NCBI database. The differential expression of *TapAPX* at different stages of wheat development *viz* seedling, tillering, stem elongation and anthesis stage was observed by qPCR analysis (Figure 1) and fold expression of 203 times of *TapAPX* at 42°C stress during anthesis stage in heat tolerant cv. Raj 3765 was observed. *TapAPX* was also upregulated at 37°C of heat stress during anthesis stage in wheat though the up-regulation was observed to be only 3.2 fold. A base level of gene expression was experienced in heat susceptible wheat cv. HD 2967 during similar stage at heat stress of 37°C & 42°C. A comparative analysis of expression of *TapAPX* at other developmental stages (seedling, tillering and stem elongation) in wheat cv. Raj3765 reflected that there was a negative fold change of expression at both 37°C and 42°C in the above mentioned stages of plant. Housekeeping gene Actin was used as constitutive control for all qPCR studies [10].

### Full length characterization of cDNA encoding for *TapAPX*gene and its expression in *E. coli*BL21 cells

**Figure 2:**
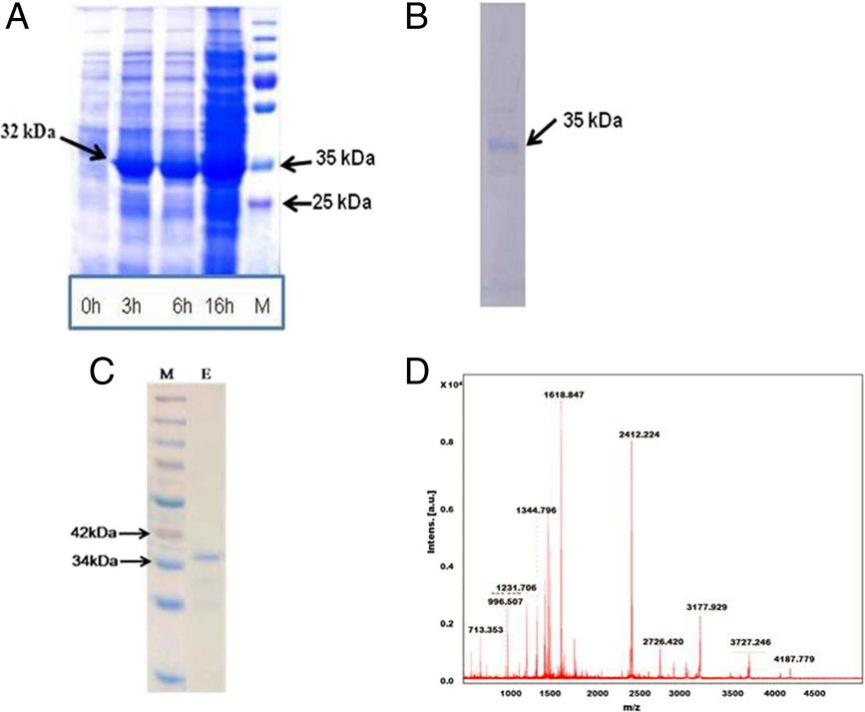
**Proteomic analysis of** ***T. aestivum pAPX*** **gene** ***.*** SDS-PAGE analysis representing the *Ta*pAPX protein expression in *E. coli* BL21 strain grown at different time periods after IPTG induction **(A)**. Western blot analysis of *Ta*pAPX protein using Anti-His antibody showing its deduced band of 32 kDa **(B)**. His-tag purification using Ni-NTA column. E- purified recombinant fusion *Ta*pAPX protein from *E. coli* BL21 (pET28a-*TapAPX)*, M-Marker **(C)**. PMF of the over-expressed APX protein using MALDI-TOF/TOF **(D)**.

Full length cDNA sequence (876 bp) of *TapAPX* gene was amplified by 5′ and 3′ RACE- PCR. The *TapAPX* cDNA amplicons obtained were cloned in pGEM-T easy vector (Promega, USA) and sequenced to get the full length *TapAPX* cDNA of 1236 bp. Nucleotide sequence showed 96 percent homology with *TapAPX* gene in Genbank databases. The obtained *TapAPX* gene sequence having an ORF of 876 bp with a 199 bp 5**′** and 161 bp 3**′** untranslated regions (UTRs) coding a protein of 292 amino acids with a predicted isoelectric point of 7.4 (http://web.expasy.org/translate/). The deduced protein had an approximate molecular weight of 32 kDa and the translated amino acid sequence showed an overall 83 to 98 percent identities with *APX* from *Hordeum vulgare* [Genbank:BAB62533], *Aegilops tauschii* [Genbank:EMT10887], *Puccinellia tenuiflora* [Genbank:AGW23429], *Oryza sativa Japonica* [Genbank:NP_001062439], *Brachypodium distachyon* [Genbank:XP_003574893]. The *TapAPX* cDNA was cloned in expression vector pET-28a(+) and transformed in *E. coli* BL21. The white colony of *E. coli* BL21 cells containing pET-28a(+)-*TapAPX* recombinant plasmid was inoculated in LB media. IPTG was added to the media for induction of 32 kDa fusion protein which was successfully expressed having similar molecular weight of barley *HvAPX*. It was also noticed that the amount of expressed protein was enhanced as the time of IPTG induction increased (0 h, 3 h, 6 h and 16 h) as evident from the intensity of band on SDS PAGE gel (Figure 2A). This confirmed that the *Ta*pAPX protein was expressed in *E. coli* as in the expected manner. The activity of *Ta*pAPX protein expression was detected in bacterial extracts by SDS PAGE showing a prominent and enriched band with an apparent size of 32 kDa.

### Western blotting, purification and PMF (Peptide mass fingerprinting) of the expressed *Ta*pAPX protein

The expression vector used has His Tag (Histidine Tag) 5**′** upstream of the cloning site. As a result, the recombinant protein has a 6X Histidine at N- terminal. To confirm the expressed recombinant protein, western blotting analysis was carried out with Anti-His antibody for hybridization to His-Tag of recombinant protein. The developed blot showed the presence of a single band of the expected size (Figure 2B). The overexpressed *Ta*pAPX protein purified using Ni-NTA column showed the presence of a single band (~35 kDa) (Figure 2C). The sequencing results obtained after PMF of the overexpressed protein band using MALDI-TOF/TOF (Matrix Assisted Laser Desorption/Ionization-Time of Flight) confirmed the *Ta*pAPX protein (Figure 2D). The sequencing results obtained after MALDI showed highest homology with a protein having molecular weight of 31832 Da.

### Heat stress tolerance in *E. coli*

**Figure 3:**
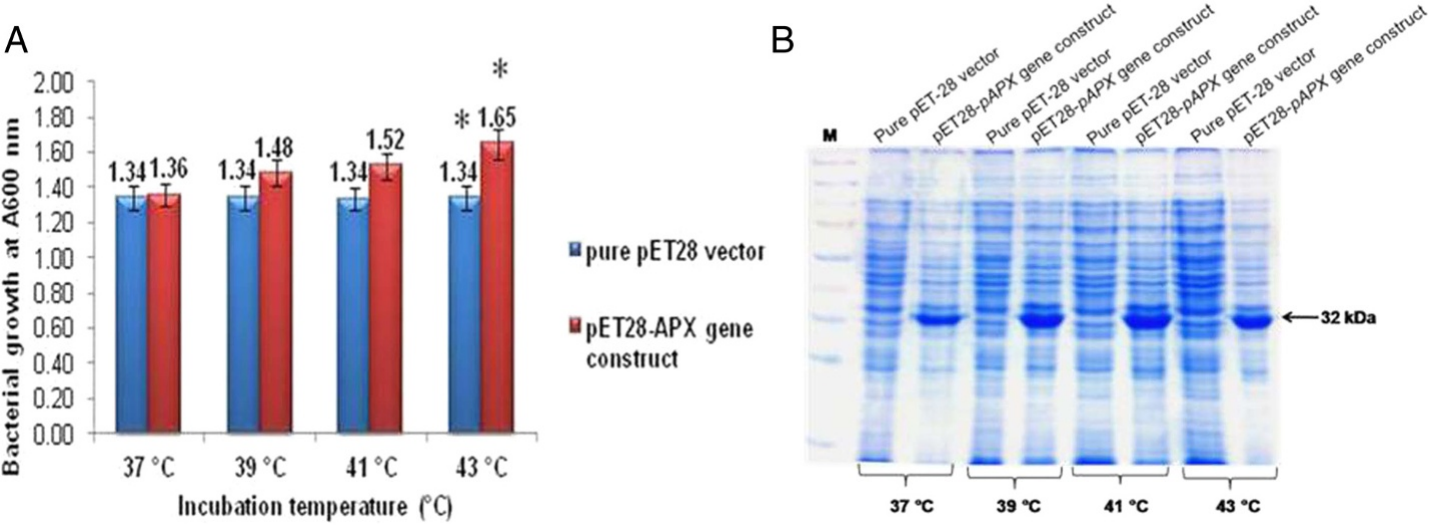
**Heat stress study of recombinant** ***E. coli*** **BL21 (pET28a-** ***TapAPX*** **)** **cells.** OD reading of *E. coli* BL21 (pET28) cells and *E. coli* BL21 (pET28-*TapAPX*) cells grown at different temperatures after IPTG induction **(A)**. SDS-PAGE analysis of total protein (10 μg) of *E. coli* BL21 (pET28) cells and *E. coli* BL21 (pET28-*T*a*pAPX*) cells subjected to heat stress, M-Marker **(B)**. *indicates significant difference as determined by simple pair wise t-test comparison (α = 0.05).

*E. coli* cultures harbouring the recombinant plasmid pET-28a-*TapAPX* grown at temperature viz 37°C, 39°C, 41°C and 43°C higher than optimum temperature for *E. coli* growth showed continuous increased growth in comparison to *E. coli* cells having pET-28a vector only, as evident by O.D. (Optical Density) at A600 of *E. coli* cultures at different temperatures (Figure 3A, Additional file 2: Table S2). Total protein from bacterial cells of *E. coli* transformed with pET-28a-*TapAPX* showed over expression of *pAPX* gene as evident on SDS-PAGE where no expression of *TapAPX* gene was observed in case of *E. coli* transformed with pET-28a vector (Figure 3B).

### *In-silico*characterization of *TapAPX*

#### Sequence analysis

**Figure 4:**
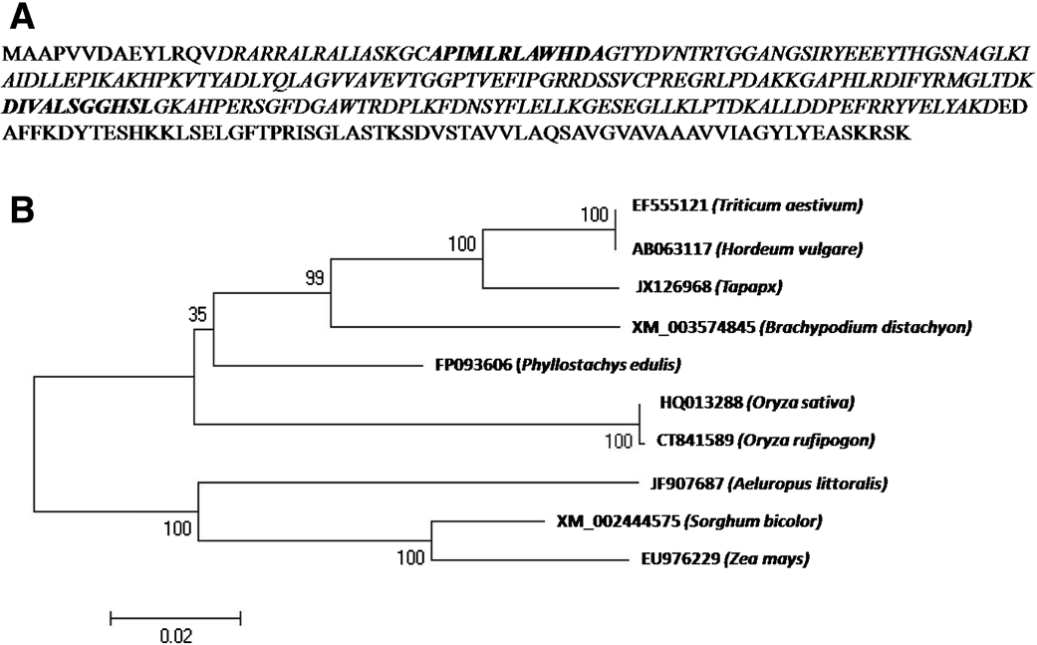
**Sequence and phylogenetic analysis.** Deduced amino acid sequences of *TapAPX* gene showing the functional sites domain (bold and italics) and the peroxidase region of *Ta*pAPX (italics) **(A)**. Phylogenetic tree analysis of *TapAPX* gene at nucleotide level from different sources **(B)**.

The phylogenetic tree constructed by using full length CDS sequences of *Ta*p*APX* gene available in NCBI database depicts that the present isolate *TapAPX* well clustered with *Triticum aestivum* [Genbank:EF555121.1] and *Hordeum vulgare* [Genbank:AB063117.1] both having 96% identity whereas only 85% identity was observed with cluster of *Aeluropus littoralis* [Genbank:JF907687.1] and *Zea mays* [Genbank:EU976229.1] (Figure 4A, B) [11]. The protein sequence of *TapAPX* subjected to PROSITE scan database revealed the presence of 2 functional sites i.e. from residue position number 31–42 and 152–162 and PFAM search database displayed the peroxidase region of *Ta*pAPX protein from 15–224. Physiochemical properties of protein obtained from ProtParam tool revealed that the present protein sequence contains 292 amino acids and has a molecular weight of 31770.3 Da with a theoretical pI of 7.74. Alanine (11.7%) followed by Leucine (10.3%) and Glycine (8.6%) were the maximum number of amino acid residues present in the protein sequence. The total number of negative (Aspartic acid + Glutamic acid) and positively charged (Arginine + Lysine) residues were 39 and 40 respectively. The instability index (II) was computed to be 31.07 and it classifies the protein as stable. The grand average of hydropathicity (GRAVY) was calculated to be -0.270 which indicates the solubility of the protein to be hydrophobic. Secondary structure of *Ta*pAPX protein generated by GOR IV method generated an alpha helix region to be of 32.65%, extended strand region of 16.84% and Random coil region of 50.52%.

#### Three dimensional structure generation

**Figure 5:**
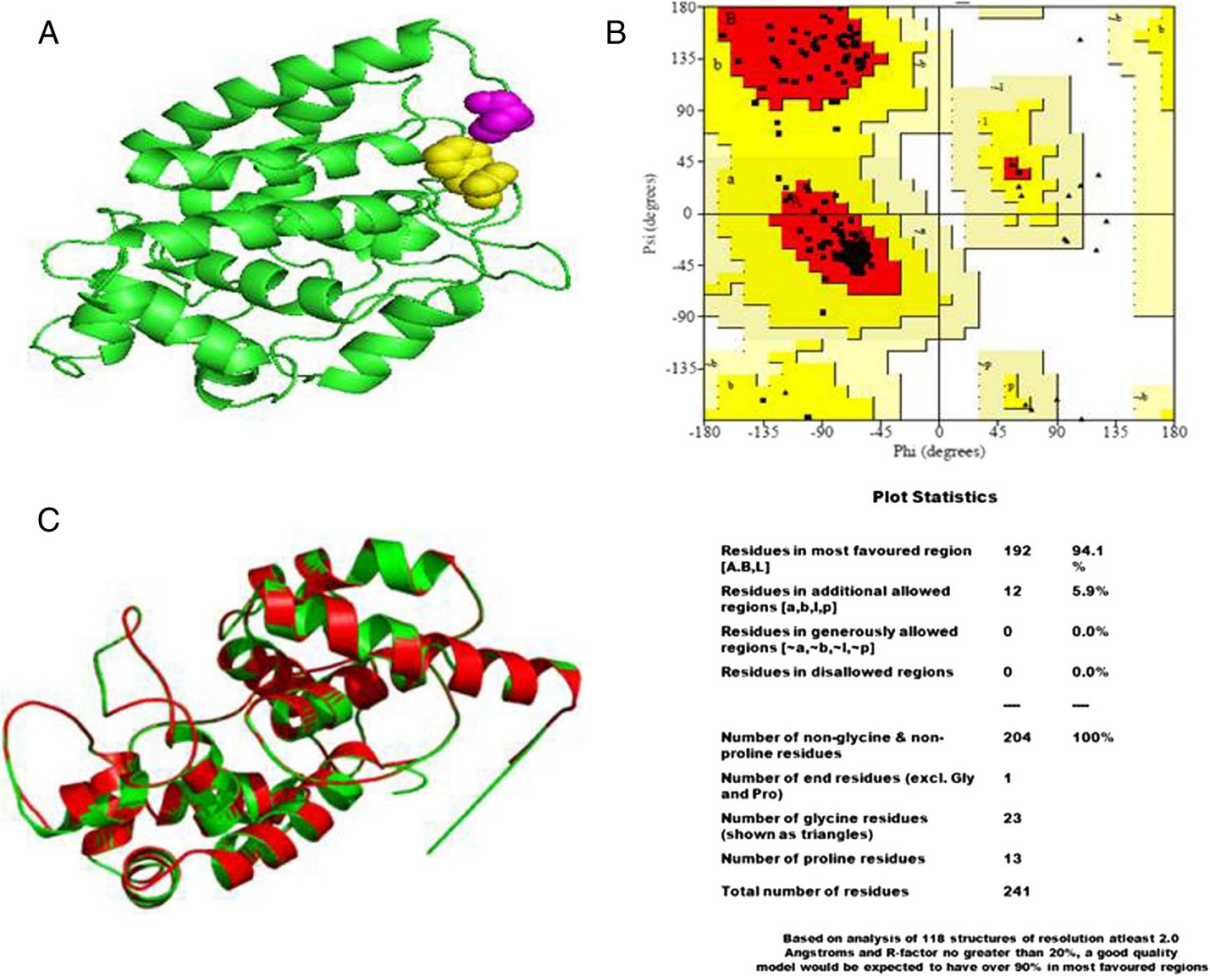
**3D structure of** ***Ta*** **pAPX protein.** Showing N-terminal (pink colour) and C-terminal (yellow colour) **(A)**. Ramachandran plot of *Ta*pAPX protein revealing 94.1% residues located in the most favored regions and 5.9% residues in semi allowed region **(B)**. Superimposed model of generated protein structure of *Ta*pAPX under study (green) against its template 2XIF (red) **(C)**.

The model of wheat *Ta*pAPX protein was generated by homology modeling using different servers. The PDB Blast analysis revealed that the protein sequence of *Ta*pAPX showed maximum identity (64%) with Ascorbate Peroxidase of *Glycine max* [PDB:2XIF_A] (http://www.rcsb.org/pdb/home/home.do). On the basis of Ramachandran plot and Verify3D program, the protein structure generated from SWISS-MODEL was selected for further analysis. Structure of *Ta*pAPX was visualized using PyMOL (Figure 5A). The PROCHECK analysis of protein revealed that no amino acid residues have phi/psi angles in the disallowed regions (Figure 5B) of Ramachandran plot which indicates that the protein is highly stable. Verify3D program showed good 3D_1D profile score of the residues i.e. 99.17% residues had an average 3D-1D score of >0.2. The QMEAN server used to find the overall quality of three dimensional structure of *Ta*pAPX protein displayed a QMEAN and QMEANZ score of 0.816 and 0.5 respectively suggesting that TapAPX protein model is acceptable. The 3D protein model has been submitted to Protein Model Data Base [PMDB:PM0079451] (http://bioinformatics.cineca.it/PMDB/). Comparative study of *Ta*pAPX model and its template 2XIF_A by iPBA webserver showed a root mean square deviations (RMSD) score of 0.16 Å as measured by average distance between the backbones of both the models (Figure 5C).

#### Active site identification and docking study

**Figure 6:**
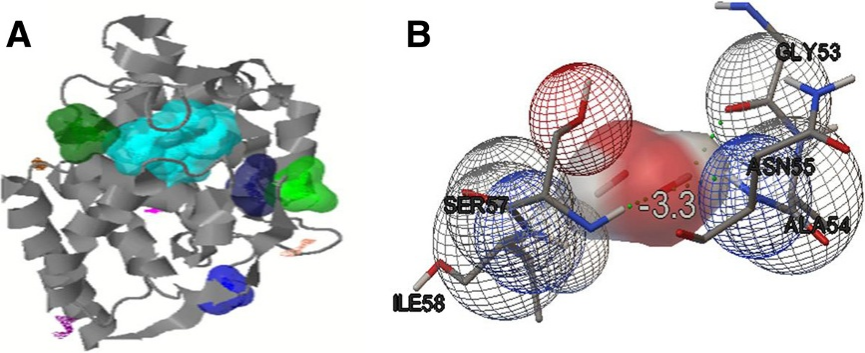
**Active sites and interaction of receptor-ligand.** Model of generated *Ta*pAPX protein showing ten active sites by Q-SiteFinder tool (in different colours) containing different amino acid residues. First five active sites shown in space fill **(A)**. Molecular interaction studies between *Ta*pAPX model and substrate H_2_O_2_ by Autodock vina software_._ Green dots represent the Hydrogen bonding between ASN55 and SER57 **(B)**.

Ten different active sites were identified (Figure 6A) in the generated *Ta*pAPX 3D protein model by Q-SiteFinder (Table 2). H_2_O_2_ ligand molecule was retrieved from pubchem database [PubChem:CID_784] for docking studies of the generated protein structure. Nine different confirmations of docking between the receptor (*Ta*pAPX) and the ligand molecule (H_2_O_2_) were obtained using Autodock vina. The best docked interaction model of *Ta*pAPX with H_2_O_2_ (hydrogen peroxide) was analyzed by Autodock tool. Each ligand represents a specific binding energy where the ligand containing lowest binding energy conformation was considered the most acceptable docking structure. The docked confirmation with lowest binding energy score i.e. -3.3 was selected for further analysis. Docking analysis clearly indicates that the ligand molecule was involved in the interaction with *Ta*pAPX model and that H_2_O_2_ formed H bond between 2 amino acid residues i.e. ASN55 and SER57 (Figure 6B).

**Table 2:** **Ten active sites of** ***Ta*** **pAPX protein model showing its different residues**

Site	Active site residues
Site 1	Lys164, Ala165, His166, Arg169, Ser170, Phe172, Trp176, Tyr187, Leu200, Leu202, Thr204, Asp205, Leu208, Tyr232, His236
Site 2	Gly43, Thr44, Tyr45, Asp46, Val47, Arg125, Gly127, Arg128, Asp141, Ile142, Phe143, Arg145, Met146
Site 3	Gly29, Cys30, Ala31, Pro32, Ile33, Leu162, Gly163, Lys164, His166, Arg169, Ala175, Pro180, Leu181
Site 4	Pro4, Asn55, Gly116, Arg117, Arg118, Ser120
Site 5	Thr44, His66, Ser68, Asn69, Pro124, Arg125, Glu126, Gly127, Arg128, Leu129, Pro130
Site 6	Glu9, Tyr10, Arg12, Gln13, Lys85, His86, Pro87, Lys88, Val89
Site 7	Thr110, Val111, , Glu112, Lys230, Thr233, Glu234
Site 8	Thr51, Gly52, Val122, Cys123, Pro124, Arg125, Arg128
Site 9	Lys151, Arg216, Tyr217, Leu220, Tyr221, Asp231
Site 10	Ile25, Gly26, Gly29, Cys30, Ala31, Pro32, Val105, Thr106, Leu181

## Discussion

For cloning of differentially expressed genes, Suppression Subtractive Hybridization (SSH) has proved to be a powerful tool for identifying abiotic stress (heat, drought, salt, nutrient deficiency etc.) responsive gene transcripts in plants [12, 13]. In our study, the thermo- tolerant wheat cv. Raj 3765 subjected to heat stress of 37°C and 42°C for different time periods (½ h, 1 h, 2 h, 4 h and 6 h) was selected as tester and normally growing cv. Raj 3765 as control. These plant groups were given heat treatment to get a wide range of heat responsive transcripts expressing at two variable high temperatures. TBARS results with the heat stressed samples showed that MDA concentration increase in case of HD2967 was in a very wide range whereas the heat tolerant cv. Raj 3765 showed MDA variation in a limited range in response to heat stress. Moreover, a rapid decrease in MDA concentration in the heat stress samples of cv. Raj3765 is suggestive of a protection mechanism against oxidative damage due to heat stress which maybe controlled by higher induced activities of antioxidant enzymes [11, 14]. From the differentially expressed 204 ESTs, which were obtained in subtractive library, *Ta*p*APX* was cloned in full length using RACE-PCR. Heat treatment results in H_2_O_2_ production and *APX* plays an important role in eliminating H_2_O_2_ using ascorbate as a specific electron donor. Expression of *APX* activity was 203 times higher in thermo-tolerant variety as compared to the susceptible one and it was also highly active in 42°C rather than 37°C. The transcript level of *TapAPX* gene increased gradually at anthesis stage, which is considered as critical developmental stage and is highly sensitive to heat stress [15]. For the qPCR studies, *Actin* gene was used as internal control, though other housekeeping genes like: Glyceraldehyde-3 phosphate dehydrogenase, 18S rRNA etc. also can be used as internal control in qPCR studies [16]. *APX* has been cloned from many other crops like cotton, *A. thaliana*, barley [17–19] and also from wheat expressing against powdery mildew disease [20]. The role of *TapAPX* in heat stress response was validated when the gene was expressed in prokaryotic system. Bacterial cells *E. coli* BL21 harbouring a recombinant plasmid over expressing the *TapAPX* gene of wheat could tolerate high temperature as evident by a gradual increase of cell density measured by O.D. as compared to cells having pET-28a vector which were sensitive to heat stress.

A heterologous expression system was used for high level expression of *TapAPX* in *E. coli* and further facilitated to obtain highly purified *Ta*pAPX protein by Ni-NTA Histidine based purification system. The purified protein could be useful for the production of protein specific antibody. Protein sequence of *Ta*pAPX over expressed in bacterial system was confirmed by peptide mass fingerprinting. The molecular interactions of *Ta*pAPX with its substrate furnished by computational analysis confirmed its strong connection to degrade ROIs such as H_2_O_2_. The CDS of *TapAPX* gene could be potentially useful for the development of heat tolerant transgenic crop plants.

The homology search comparative modeling and docking studies finally validated the functional correlation between enzyme *Ta*pAPX and its substrate H_2_O_2_. The refined *Ta*pAPX 3D structure was successfully generated and its active site residues were identified. 3D structure provides the useful information related to molecular function and identification of active sites [21]. PDB PSI- BLAST was searched for finding its template showing maximum identity of 64% that can be considered as a good score to start modeling. It was observed that two distinct amino acid residues viz. Asn and Ser which are potentially involved to recover the normal physiological metabolism against abiotic stress [22, 23]. Further detailed molecular biology work on the expression of *TapAPX* in *Arabidopsis* plant and common wheat is going on in our laboratory which would provide a valuable work in understanding the mechanism of heat stress tolerance in wheat.

*In-silico* based approach and characterization of *TapAPX* at nucleic acid and proteomics level revealed the membrane bound nature of this gene. The nucleotide sequence of *TapAPX* and its deduced amino acid sequence analysis obtained after PMF (Peptide Mass Fingerprinting) of the differentially expressed protein bands on SDS-PAGE revealed that it belongs to peroxisomal type of peroxidase. *In-silico* characterization of this gene was carried out by homology BLAST search, multiple sequence alignment, construction of phylogenetic tree, 3D structure and active sites generated by homology modeling and thereby enzyme- substrate interaction study by docking analysis. The docking analysis by Autodock vina tool revealed that hydrogen bonding between H_2_O_2_ with Asn and Ser residues of *Ta*pAPX and may cause its breakdown during biochemical reaction. The recombinant *Ta*pAPX protein produced in *E. coli* BL21 cells was able to rescue cells growing at higher temperature (43°C) as compared to control. The changes in cell growth (in terms of O.D.) in comparison with its control was found to be statistically significant (simple pair wise t-test) when cells were exposed upto 43°C stress where it was not changed distinctly for other low temperature stress conditions. In this study, the heat stress was maximized up to 43°C for bacterial cells by taking into consideration heat stress imposition at 42°C to the plants just before the SSH library construction. However, it is possible that more significant changes may be noticed, if the bacterial cells are exposed to temperature stresses of above 43°C and upto a sub lethal temperature. Real time analysis have also shown a very high level gene expression in terms of fold change (F.C.-203) when plants were exposed to heat stress at 42°C. *In vitro* results together with *in-silico* studies confirm the high level of enzyme activity of this gene in order to improve tolerance under abiotic stress and it indicates that the *TapAPX* gene plays a leading role in mediating overlapping cellular processes especially heat and oxidative stress. This finding will help us to validate not only abiotic stress but also biotic stress response of this enzyme in model plant systems and as well as improvement of genetic background of several crop plants susceptible to abiotic stresses by implying transgenic technologies.

## Conclusions

Complete CDS of *TapAPX* from thermotolerant wheat cv. Raj3765 was isolated, cloned sequenced and characterized (*in-sili*co) for the first time in Indian bread wheat. qPCR studies confirmed the role of *TapAPX* gene in thermo tolerance in wheat. The over expressed *Ta*pAPX protein was functionally validated in *E. coli* by western blot and MALDI. Biological validation of *TapAPX* gene in prokaryotic system was confirmed by growth at high temperature of recombinant *E. coli* cells harbouring wheat *TapAPX* gene showing significant changes subjected to stress of 43°C. Ramachandran plot, protein 3D structure and docking analysis have given a deep understanding of *TapAPX* gene.

## Methods

### Plant materials, heat stress treatment, lipid peroxidation assay and SSH cDNA library construction

Heat tolerant wheat (*Triticum aestivum*) cv. Raj3765 plants and heat susceptible wheat cv. HD2967 [10] plants were grown in National Phytotron Facility, IARI, New Delhi under a light period of 16 h at ±25°C and light intensity of 350 μ molm-2 s-1 and dark period of 8 h [24]. Heat treatment was given to plants at anthesis stage at 42°C for different time periods (½ h, 1 h, 2 h, 4 h and 6 h). Lipid peroxidation assay was performed according to the TBARS (Thiobarbituric Acid Reacting substances) method [25]. Non specific absorbance of the extract at 600 nm was subtracted from the 532 nm readings to find out the absolute amount. Total RNA from heat stressed and heat unstressed plants were extracted using Spectrum™ Plant Total RNA Kit (Sigma, USA). cDNA was prepared from 1 μg of total RNA using SMART PCR cDNA synthesis kit (Clontech laboratories, USA) according to manufacturer’s protocol. The forward and reverse libraries were constructed using PCR select cDNA subtractions kit (Clontech laboratories, USA). The expressed secondary PCR amplified products were cloned into pGEM-T easy vector (Promega, USA). The obtained clones of forward and reverse libraries were sequenced in an automated sequencer (ABI Prism 310, Applied Biosystems, USA). All the good EST sequences were assembled into contigs and singlets by using CAP3 sequence assembly program (http://doua.prabi.fr/software/cap3). The assembled sequences representing unigene data sets were further analyzed for identity search (BLASTX) to the NCBI BLAST program by using BLAST2GO program (http://www.blast2go.com/b2ghome) for identifying heat stress responsive genes.

### Real time PCR of *TapAPX*transcripts

Plants at different developmental stages viz. seedling, tillering, stem elongation and anthesis stages were subjected to heat stress treatment in 37°C and 42°C for different time intervals i.e. ½ h, 1 h, 2 h, 4 h and 6 h. Similar heat stress was also imposed to heat susceptible wheat cv. HD2967 at anthesis stage [10] for checking the varietal differences. The stressed and unstressed plant samples were harvested, immediately frozen in liquid N_2_ and stored at -80°C for downstream experiments. Total RNA was isolated using Spectrum™ Plant Total RNA Kit (Sigma, USA) as per manufacturer’s instructions. The cDNA synthesis was carried out from the isolated RNA by using SuperScript™ III First-Strand Synthesis System (Invitrogen, USA). The qPCR reaction was performed with the synthesized cDNA as template. Based on the sequence information of EST of the forward SSH library, qPCR primers for *TapAPX* was designed (Table 3). The reaction [Lightcycler 480 SYBR green Master mix, 2X-10 μl (Roche, USA); PCR primers (Forward and Reverse), 10 mM-1 μl each; cDNA template, 40 ng/μl-5 μl and PCR grade water-3 μl] was carried out using LightCycler® 480 II System (Roche, USA). For endogenous control, constitutively expressed Actin gene was used. All the reactions were done in triplicate.

**Table 3:** **Primer pair A: To amplify actin gene, B: Real time primer for** ***TapAPX*** **gene, C: To amplify full length** ***TapAPX*** **gene with restriction sites shown in italics**

S. no.	Gene	Primer sequence	Sequence amplified
A	*Actin*	F 5′ GAAGCTGCAGGTATCCATGAGACC3′	151 bp
		R 5′ AGGCAGTGATCTCCTTGCTCATC3′	
B	*TapAPX*	F 5′ GATGCTAAGAAAGGCGCACCACAT3′	124 bp
		R 5′ AGGCACATCCTGAAAGGTCTGGTT3′	
C	*TapAPX*	F 5′ CGC*GGATCC*ATGGCGGCTCCGGTGGTGGACG3′	876 bp
		R 5′C*GAGCTC*TTACTTGCTCCTCTTGGAAGCCTCGTACAG3′	

### RACE (rapid amplification of cDNA ends) PCR of *TapAPX*gene and heterologous protein expression in *E. coli*

The 5**′** and 3**′** RACE PCR (Rapid amplification of cDNA ends) were performed in separate reactions to obtain full length sequence of *TapAPX* gene by using SMARTer™ RACE cDNA Amplification Kit (Clontech laboratories, USA). The fragments obtained after 5**′** and 3**′** RACE-PCR were cloned independently in pGEM-T Easy vector (Promega, USA) and thereafter sequenced to get full length cDNA sequence along with 5**′** upstream and 3**′** downstream sequences.

Specific primers (Table 3) were designed for cloning of *TapAPX* full length cDNA in pET-28a expression vector (Novagen, USA). The oligonucleotide of the primer sequences were designed in a manner to introduce BamHI site just before the start codon ATG and SacI site just after the stop codon (TAA). Using suitable concentration of the designed primers (10 mM, 0.5 μl each), dNTPs of (25 mM) 0.25 μl, MgCl_2_-1.25 μl, DNA polymerase- 0.25 μl and DNA polymerase buffer (10×)- 2.5 μl, full length coding *TapAPX* sequence was PCR amplified using total cDNA (200 ng) as a template. The amplified PCR product was purified using QIAquick PCR purification kit (Qiagen, USA). 1 μg of PCR purified product of (*TapAPX*) was digested with 1 μl each of 20 U/μl of restriction enzymes BamH1 and Sac1 (NEB, USA) in a reaction of 20 μl, the vector pET-28a (500 ng) digested with same set of restriction enzymes. The digestion reaction was carried out at 37°C for 3 hours. The digested PCR product was cloned in pET-28a vector using T4 DNA ligase, the ligated product was transformed in *E. coli* DH5α and recombinant clones were selected on LA plates supplemented with antibiotic Kanamycin 30 μg/ml. The positive clones were further screened by colony PCR using gene specific primers of *TapAPX*. Sequencing of the clone having *TapAPX* gene was carried out using T7 promoter primer to reconfirm the presence of *TapAPX* gene along with the presence of 6X His-tag at the 5**′** upstream of the expression vector pET-28a. The expression study of *TapAPX* gene in prokaryotic system was done by transforming the pET-28a-*TapAPX* recombinant plasmid in *E. coli* BL21 cells (Novagen, USA) using heat shock method [26]. The positive clone obtained on selection media (LA + 30 μg/ml Kanamycin) was inoculated in LB supplemented with 30 μg/ml kanamycin and incubated at 37°C. Isopropyl β-D thiogalacto pyranoside (IPTG), an inducer of T7 promoter in pET-28a vector, was added at final concentration of 1 mM when O.D of the culture reached an absorbance of 0.5 at 600 nm. *TapAPX* which is now under the control of T7 promoter in pET-28a vector, samples were collected at 0, 3 h, 6 h and 16 h after induction was given. The samples were resuspended in protein extraction buffer (100 mM Tris–HCl, pH-7.5, 1 mM of PMSF in isopropanol, 10 mM EDTA (Ethylene Diamine Tetra Acetic acid) and 1.6 μg/ml of lysozyme (final concentration) and kept on ice for 1 h. Total protein was quantified using Nanodrop spectrophotometer (Thermo Scientific NanoDrop 2000C Technologies, Wilmington, USA) and 20 μg of total protein was loaded on two separate 12% SDS-PAGE gel [27], one gel was used for coomassie staining to visualize the protein bands and other for western blotting to confirm the identity of protein under study.

### Western blotting of *Ta*pAPX protein and purification

The *Ta*pAPX protein from the SDS-PAGE was transferred to the PVDF membrane (BIO-RAD, USA) using Mini Trans-Blot® cell MTB module (BIO-RAD, USA) using a constant supply of 45 V for 1 h. The presence of pre-stained marker on the membrane confirmed the transfer process. The membrane was then incubated in blocking solution 5% BSA (Bovine serum albumin) in TSW buffer (10 mM Tris–HCl, pH-7.4, 0.02% SDS, 0.9% NaCl, 0.1% Triton X-100) on a gyro-rotary shaker at room temperature for 1 h. Further the membrane was incubated with anti-His-tag antibody (Mouse monoclonal Antibody) (Abm, Canada) at a dilution of 1:4000 for 1 h. Three washes of 10 min each was given with TSW buffer followed by incubation with Alkaline Phosphatase Conjugated Affinity Purified Antimouse secondary antibody (Abm, Canada) with same dilution and incubated for 1 h. After washing with TSW buffer for 3 times (10 min each), the membrane was developed using NBT/BCIP substrate solution. The presence of single band at appropriate location confirmed the presence of recombinant protein. For protein purification, the total protein was extracted from overnight grown *E. coli* BL21 cell containing pET28a-*TapAPX* construct by using Total Protein Extraction kit (G-Biosciences, St. Louis, USA). 500 μl of the extracted total protein was loaded directly on His SpinTrap columns (GE Healthcare, Amersham, UK) containing Ni Sepharose High Performance medium for perfectly binding of Histidine tagged protein. The purification steps were followed according to manufacturer’s protocol and the purified protein was further checked on 12% SDS-PAGE.

### PMF (Peptide mass fingerprinting) of the expressed protein

The SDS-PAGE gel selected for Coomassie staining having over-expressed *Ta*pAPX protein band was sliced out using a sharp scalpel. The gel slice was diced to small pieces and placed in eppendorf tubes. The gel pieces were destained using destaining solution for 10 min intervals (3–4 times) by vortexing untill the gel pieces become translucent white. The gel pieces were dehydrated using acetonitrile and Speedvac till complete dryness, after that rehydration was done with DTT (Dithiothreitol) and incubated for 1 h. After incubation the DTT solution was removed which was replaced with Iodoacetamide and incubated for 45 min. The supernatant was removed and the gel pieces were incubated with ammonium bicarbonate solution for 10 min. Again supernatant was removed and the gel pieces were dehydrated with acetonitrile for 10 min and dried using speedvac. Trypsin solution was added to gel pieces and incubated overnight at 37°C. After incubation the supernatant, which is now having peptides, was transferred to fresh eppendorff tubes. The gel pieces were extracted thrice with extraction buffer and the supernatant was collected each time into the same eppendorff tube and then given Speedvac till complete dryness [28]. The dried pepmix was suspended in TA buffer. The peptides obtained were mixed with (α-cyano-4-hydroxycinnamic acid) HCCA matrix in 1:1 ratio and the resulting 2 μl mix was spotted directly onto the MALDI plate. After air drying the sample, it was analyzed on the MALDI TOF/TOF ULTRAFLEX III instrument (Bruker, Germany) and further analysis was done with FLEX ANALYSIS SOFTWARE for obtaining the PEPTIDE MASS FINGERPRINT (PMF). The masses obtained in the peptide mass fingerprint were submitted for Mascot search in “plant” database for identification of the protein.

### Heat stress tolerance study in *E. coli*

The *E. coli* BL21 cells containing pET28a-*TapAPX* construct was used for heat stress tolerance study. The initially grown bacterial cell samples at 37°C were taken for IPTG induction (1 mM) and thereafter kept at 37°C, 39°C, 41°C and 43°C for 6 h. *E. coli* BL21 cells with pET-28a vector only were used as negative control. The O. D. at A_600_ was measured and the statistical analysis was done using simple pair wise t-test in comparison to respective control at an α level of 0.05. The total cell protein (10 μg each) from bacterial samples heat stressed at different temperatures was weighed down in each well on a 12% SDS-PAGE gel to check the expression variation of recombinant protein.

### *In-silico*characterization of *TapAPX*gene

#### Sequence analysis

The cloned CDS sequence of *TapAPX* gene was searched for homology with NCBI database by BLASTN and its translated protein sequence for the complete ORF was retrieved from NCBI database by BLASTP search [29]. The complete sequence of present isolate was compared with reported nine isolates in different monocots available in GenBank. Multiple alignment and sequence identity matrix of the sequences of *TapAPX* gene was carried out using Clustal Omega program (http://www.ebi.ac.uk/Tools/msa/clustalo/) [30]. Phylogenetic analysis based on neighbor-joining method was conducted in MEGA4 (http://www.megasoftware.net/mega4/mega.html) [31] to investigate the ancestral relationships and closely related species. The protein domain functional analysis of *Ta*pAPX protein sequence was searched by PROSITE (http://prosite.expasy.org/) [32], PFAM (http://pfam.xfam.org/) [33] and conserved domain was searched by CDD (http://www.ncbi.nlm.nih.gov/Structure/cdd/cdd.shtml) [34]. The physicochemical properties of *Ta*pAPX protein were analyzed by ProtParam tool (http://web.expasy.org/protparam/) [35]. The secondary structure of *Ta*pAPX protein was genrated by GOR IV server (http://npsa-pbil.ibcp.fr/cgi-bin/npsa_automat.pl?page=npsa_gor4.html) [36].

#### Three dimensional structure generation

For the modeling of three dimensional structure, a suitable template was searched by using PDB PSI-BLAST (Position-Specific Iterated BLAST) [37]. Construction of three dimensional structures by using different homology modeling servers like Phyre2 (http://www.sbg.bio.ic.ac.uk/phyre2/html/page.cgi?id=index) [38], ESyPred3D (http://www.unamur.be/sciences/biologie/urbm/bioinfo/esypred/) [39], Protein Structure Prediction Server (PS)^2^ (http://ps2.life.nctu.edu.tw/) [40], SWISS-MODEL (http://swissmodel.expasy.org/interactive) [41], Jigsaw (http://bmm.cancerresearchuk.org/~3djigsaw/) [42] and I-Tasser (http://zhanglab.ccmb.med.umich.edu/I-TASSER/) [43] was performed to find out the best one. By using SAVES (Structural Analysis and Verification Server) (http://services.mbi.ucla.edu/SAVES/), the conformations of generated models were inspected by the Phi/Psi Ramachandran plot obtained from PROCHECK server [44] and Verify_3D [45] was used to find the acceptable average 3-D ID score. The quality of *Ta*pAPX protein models was checked by using Qualitative Model Energy Analysis (QMEAN) server (http://swissmodel.expasy.org/qmean/cgi/index.cgi) [46]. On the basis of model stability, best model was selected from SWISS-MODEL server. The PyMOL (http://www.pymol.org/) [47] software was used to visualize the 3D structure and the iPBA webserver (http://www.dsimb.inserm.fr/dsimb_tools/ipba/) [48] was used for superimposing the generated model with its template model.

#### Active site identification and docking study

The identification of active sites of *Ta*pAPX protein structure was obtained from Q-SiteFinder tool (http://www.bioinformatics.leeds.ac.uk/qsitefinder) [49]. Docking was used to identify the specific active sites on protein where receptor- ligand interaction occurs by Autodock vina 1.1.2 (http://vina.scripps.edu/index.html) [50]. The structure of H_2_O_2_ (hydrogen peroxide) ligand molecule available in PubChem site (http://pubchem.ncbi.nlm.nih.gov/) [51] of NCBI database in SDF (Sql Database File) format and the conversion of ligand to PDB format was done using the Open babel software (http://openbabel.org/wiki/Main_Page) [52]. The file format conversion of the receptor and ligand structures from PDB to PDBQT was performed by using Autodock tool (ADT) (http://autodock.scripps.edu/resources/adt) [53]. A grid-box was generated to cover the entire protein structure so that the ligand molecule moves freely. The dimension of grid-box was kept as 22 Å × 24 Å × 28 Å and spacing of grid point set at 1 Å.

### Availability and requirements

The software and bioinformatics tools used in this manuscript are mentioned above along with hyperlink.

## Electronic supplementary material

Additional file 1: Table S1: Representative ESTs of forward 42°C heat stress SSH library from wheat. Assembled ESTs displaying a total number of 101 unigenes consisting of 29 contigs (EST. 1-29) and 71 singlets (EST. 30-101). (DOC 137 KB)

Additional file 2: Table S2: Table showing the O.D. at A_600_ (in replicate of 4) at different temperatures and their average. (DOCX 13 KB)

## Abbreviations

H_2_O_2_: Hydrogen peroxide
SDS-PAGE: Sodium dodecyl sulfate polyacrylamide gel electrophoresis
Ni-NTA: Nickel-nitrilotri acetic acid
cv: Cultivar
Da: Dalton
A_600_: Absorbance at 600 nm
NCBI: National centre for biotechnology information
ENA: European Nucleotide Archive.
