# Supplementary material for: Molecular cloning and in-silico characterization of high temperature stress responsive pAPX gene isolated from heat tolerant Indian wheat cv. Raj 3765
Source: BMC Res Notes. 2014 Oct 10;7:713. doi: 10.1186/1756-0500-7-713 (PMC4209082; doi:10.1186/1756-0500-7-713)
Supplement: Supplementary file 1 — Additional file 1: Table S1: Representative ESTs of forward 42°C heat stress SSH library from wheat. Assembled ESTs displaying a total number of 101 unigenes consisting of 29 contigs (EST. 1-29) and 71 singlets (EST. 30-101). (DOC 137 KB) [file 13104_2014_3252_MOESM1_ESM.doc]

**Additional file 1: Table S1: Representative ESTs of forward 42°C heat stress SSH library from wheat. Assembled ESTs displaying a total number of 101 unigenes consisting of 29 contigs (EST. 1-29) and 71 singlets (EST. 30-101).**

| EST No. | Accession No. | E- Value | Identity (%) | Best match in NCBI database |
| --- | --- | --- | --- | --- |
|  | BAH19934 | 5.98E-29 | 84 | Leucine-rich repeat family protein |
|  | AFE89428 | 9.47E-24 | 100 | Abscisic stress ripening protein |
|  | ACA30301 | 6.23E-55 | 96 | Senescence-associated protein |
|  | AEP95746 | 1.52E-139 | 96 | Glycine dehydrogenase mitochondrial |
|  | EXC17323 | 5.27E-44 | 82 | Pi-plc x domain-containing protein at5g67130-like |
|  | ACG32188 | 7.92E-28 | 100 | Tubulin beta-4 chain |
|  | XP_008383145 | 1.52E-19 | 100 | Peroxisomal -dienoyl- reductase-like |
|  | ACA58354 | 2.43E-16 | 100 | Ribulose bisphosphate carboxylase oxygenase activase chloroplastic-like |
|  | XP_008228097 | 3.73E-45 | 85 | Muscle m-line assembly protein unc- isoform 2 |
|  | AHV84769 | 6.15E-84 | 100 | Beta- partial |
|  | XP_002525821 | 3.08E-16 | 84 | Metallothionein-like protein |
|  | XP_003518775 | 1.53E-22 | 89 | Calcium-dependent phosphotriesterase superfamily protein |
|  | ACZ56426 | 1.89E-56 | 97 | 2-cys peroxiredoxin chloroplastic |
|  | XP_004161948 | 1.95E-41 | 97 | Protein translocase subunit chloroplastic-like |
|  | XP_006447588 | 9.52E-28 | 91 | Aspartic protease isoform 2 |
|  | XP_008350020 | 2.02E-101 | 94 | Protochlorophyllide chloroplastic |
|  | XP_002274753 | 2.13E-14 | 77 | C2 domain-containing family protein |
|  | AAY83342 | 1.22E-17 | 85 | Non-specific lipid transfer protein precursor |
|  | XP_004170962 | 1.29E-142 | 100 | Chlorophyll a-b binding protein chloroplastic-like |
|  | KDO77565 | 3.84E-54 | 95 | Nad -binding rossmann-fold superfamily protein isoform 1 |
|  | XP_006452683 | 2.58E-68 | 98 | Fructose-bisphosphate cytoplasmic isozyme 1 |
|  | XP_007219510 | 9.11E-04 | 68 | Hypothetical protein PRUPE_ppa022423mg, partial |
|  | EXB97676 | 6.04E-45 | 92 | Very-long-chain enoyl- reductase-like |
|  | XP_007132735 | 1.23E-61 | 96 | Senescence-associated protein |
|  | EXC04205 | 5.82E-31 | 87 | Cysteine proteinase rd19a-like |
|  | BAJ34193 | 1.12E-35 | 98 | Glycine dehydrogenase |
|  | CDO99712 | 2.16E-46 | 89 | Thiazole biosynthetic enzyme |
|  | AFV66577 | 3.03E-53 | 97 | Peroxidase 63 |
|  | XP_004302891 | 4.58E-52 | 92 | Golgin candidate 5 |
|  | XP_002303472 | 1.37E-55 | 95 | Urea-proton symporter dur3-like |
|  | XP_002532149 | 5.19E-66 | 93 | Ribulose bisphosphate carboxylase small chloroplast |
|  | ADM74103 | 1.23E-25 | 98 | Glutathione peroxidase-like partial |
|  | EXB40147 | 4.04E-86 | 92 | Core-2 i-branching beta- -n-acetylglucosaminyltransferase family |
|  | [**BAB62533**](http://www.ncbi.nlm.nih.gov/protein/15080682?report=genbank&log$=prottop&blast_rank=1&RID=YHGU5ETT01R) | **7.5E-106** | **97** | **[Peroxisome type ascorbate peroxidase](http://blast.ncbi.nlm.nih.gov/Blast.cgi#alnHdr_15080682)** |
|  | XP_007162139 | 2.56E-10 | 78 | Probable 2-oxoglutarate-dependent dioxygenase aop1 |
|  | EXB85813 | 2.27E-48 | 77 | E3 ubiquitin-protein ligase cip8-like |
|  | CDP07384 | 2.27E-50 | 96 | Endoplasmin homolog |
|  | KDP46712 | 1.29E-13 | 94 | Histone h1-like |
|  | XP_003623947 | 4.67E-07 | 68 | Trypsin inhibitor |
|  | XP_003604374 | 1.43E-42 | 97 | Post-illumination chlorophyll fluorescence increase protein |
|  | XP_006602121 | 3.27E-69 | 98 | Ribosomal rna methyltransferase nop2-like |
|  | XP_008226284 | 1.87E-30 | 91 | Fad nad -binding oxidoreductase family protein isoform 1 |
|  | EXB26577 | 5.82E-21 | 84 | Photosystem ii reaction center w chloroplastic-like |
|  | AHI54562 | 3.68E-61 | 89 | Peptidyl-prolyl cis-trans isomerase cyp20- chloroplastic-like |
|  | XP_004309907 | 2.73E-72 | 88 | F-box kelch-repeat protein at3g23880-like |
|  | EXB56921 | 8.89E-83 | 96 | Per1-like family protein isoform 2 |
|  | XP_002298285 | 2.12E-22 | 88 | Pentatricopeptide repeat-containing protein at3g59040-like |
|  | XP_007132206 | 1.96E-16 | 72 | Cell wall-associated hydrolase |
|  | XP_006436942 | 1.33E-40 | 100 | ATP-dependent Clp protease A |
|  | EXB82271 | 3.95E-17 | 77 | Target of MYB protein 1-like isoform x1 |
|  | EXC33396 | 7.77E-86 | 98 | 60s ribosomal protein l7a-like |
|  | KCW85429 | 1.54E-08 | 83 | Hypothetical protein |
|  | XP_008337734 | 1.10E-42 | 100 | Nuclease harbi1 |
|  | BAK20223 | 2.01E-76 | 98 | Probable aquaporin pip2-5 |
|  | XP_004962578 | 2.54E-15 | 95 | Photosystem i reaction center subunit xi |
|  | XP_008374347 | 7.04E-56 | 93 | Programmed cell death protein 4-like |
|  | KDP41712 | 1.74E-57 | 86 | Subtilisin-like protease |
|  | AGL07710 | 1.45E-104 | 98 | CBL-interacting serine threonine-protein kinase 6-like |
|  | XP_006374461 | 6.03E-10 | 76 | Chlorophyll a-b binding protein chloroplastic |
|  | AES77606 | 1.84E-11 | 76 | Cell wall-associated hydrolase |
|  | EME49247 | 3.02E-55 | 85 | Arm repeat-containing protein |
|  | XP_003535922 | 2.13E-63 | 98 | Magnesium chelatase h subunit |
|  | KDP31997 | 2.67E-12 | 84 | R2r3-myb transcription factor |
|  | EXB54247 | 3.50E-16 | 72 | Probable wrky transcription factor 70-like |
|  | XP_008226278 | 2.06E-52 | 93 | Translation initiation factor if- chloroplastic-like |
|  | CAH59426 | 7.77E-36 | 78 | Aleurain-like protease |
|  | XP_007201239 | 6.07E-121 | 98 | Chlorophyll a-b binding protein chloroplastic-like |
|  | XP_008372267 | 2.75E-55 | 100 | U6 snrna-associated sm-like protein lsm2 |
|  | EXC17257 | 2.57E-10 | 73 | Leucine-rich repeat family |
|  | AAY34909 | 1.94E-75 | 98 | Enolase |
|  | EXB90901 | 2.59E-61 | 89 | Probable pectinesterase pectinesterase inhibitor 6-like |
|  | XP_003633016 | 6.51E-55 | 95 | Shaggy-related protein kinase eta-like |
|  | XP_007028641 | 5.69E-76 | 98 | Calcium-binding ef hand family protein |
|  | ADV04050 | 5.52E-23 | 96 | Peptidyl-prolyl cis-trans partial |
|  | EXB35427 | 4.46E-39 | 97 | Villin 2 family protein |
|  | CBI23349 | 6.12E-58 | 91 | Beta-glucosidase 42-like |
|  | ACJ85643 | 5.20E-130 | 98 | Polyubiquitin |
|  | CAN67413 | 6.91E-119 | 98 | Aldolase-type tim barrel family protein isoform 1 |
|  | KCW86839 | 8.94E-44 | 96 | Sphinganine c -monooxygenase 1-like |
|  | EXB37318 | 1.15E-121 | 98 | Dna repair helicase xpb1 |
|  | EXB29854 | 2.71E-63 | 88 | Sieve element occlusion a |
|  | XP_008238400 | 1.44E-63 | 78 | Phospholipase a1- chloroplastic-like |
|  | XP_003633824 | 5.85E-40 | 70 | Cytochrome p450 86b1-like |
|  | NP_001105541 | 2.93E-11 | 88 | Acidic endochitinase |
|  | XP_003537123 | 4.44E-23 | 100 | Alcohol dehydrogenase class-3-like |
|  | KDP33847 | 8.51E-123 | 97 | Lycopene epsilon cyclase |
|  | CBI26116 | 6.19E-58 | 83 | Inactive beta-amylase 9 |
|  | XP_008370769 | 2.87E-77 | 94 | Gdsl esterase lipase at5g33370-like |
|  | XP_002891244 | 1.69E-107 | 98 | Glyceraldehyde-3-phosphate dehydrogenase |
|  | XP_007206187 | 1.25E-16 | 80 | Hypothetical protein PRUPE_ppa013887mg |
|  | ADZ54783 | 1.76E-77 | 92 | Flavonoid 3 -hydroxylase |
|  | AFV93496 | 1.32E-39 | 98 | Rubisco activase |
|  | ACY30440 | 1.12E-26 | 96 | Polyadenylate-binding protein 2-like |
|  | XP_008233330 | 1.54E-16 | 72 | PREDICTED: uncharacterized protein LOC103332373 |
|  | AAV74404 | 3.02E-20 | 95 | Oxygen-evolving enhancer protein 3-1 |
|  | CBI15803 | 2.25E-147 | 100 | 26s protease regulatory subunit 6b homolog |
|  | CBI21901 | 1.35E-62 | 69 | Transcription activator glk1-like |
|  | XP_008340885 | 1.93E-22 | 95 | Probable protein phosphatase 2c 35 |
|  | EXC21996 | 6.26E-21 | 96 | Rhamnose biosynthesis 1 isoform 1 |
|  | XP_007049410 | 2.27E-35 | 91 | Ring u-box superfamily |
|  | XP_006837986 | 1.04E-08 | 64 | PG1 protein |
