# Supplementary material for: Molecular cloning and in-silico characterization of high temperature stress responsive pAPX gene isolated from heat tolerant Indian wheat cv. Raj 3765
Source: BMC Res Notes. 2014 Oct 10;7:713. doi: 10.1186/1756-0500-7-713 (PMC4209082; doi:10.1186/1756-0500-7-713)
Supplement: Supplementary file 2 — Additional file 2: Table S2: Table showing the O.D. at A600 (in replicate of 4) at different temperatures and their average. (DOCX 13 KB) [file 13104_2014_3252_MOESM2_ESM.docx]

|  | **Stress given at temperature** | | | |
| --- | --- | --- | --- | --- |
|  | **37 °C** | **39 °C** | **41 °C** | **43 °C** |
| **pure pET28 vector** | 1.352 | 1.362 | 1.407 | 1.34 |
|  | 1.382 | 1.356 | 1.342 | 1.4 |
|  | 1.293 | 1.313 | 1.34 | 1.3 |
|  | 1.34 | 1.34 | 1.34 | 1.34 |
| Average | 1.34 | 1.34 | 1.256 | 1.32 |
| **pET28-*TapAPX* gene construct** | 1.364 | 1.5 | 1.52 | 1.62 |
|  | 1.43 | 1.42 | 1.46 | 1.77 |
|  | 1.28 | 1.52 | 1.59 | 1.55 |
|  | 1.358 | 1.480 | 1.523 | 1.647 |
| Average | 1.36 | 1.48 | 1.52 | 1.65 |

Additional file 2: Table S2 Table showing the O.D. at A_600_  (in replicate of 4) at different temperatures and their average
